# Dual-Functional Antenna Sensor for Highly Sensitive and Selective Detection of Isopropanol Gas Using Optimized Molecularly Imprinted Polymers

**DOI:** 10.1021/acssensors.4c03393

**Published:** 2025-02-20

**Authors:** Mohammad Mahmudul Hasan, Onur Alev, Michael Cheffena

**Affiliations:** † Faculty of Engineering, Norwegian University of Science and Technology (NTNU), Gjøvik 2815, Norway; ‡ Department of Physics, Gebze Technical University, 41400 Gebze, Kocaeli, Turkey

**Keywords:** MIP, CNT, VOC, molecularly imprinted polymer, carbon nanotubes, antenna sensor, microwave sensor, dual-functional, gas sensor, wireless sensor network, isopropanol, selective, sensitive, volatile organic compounds

## Abstract

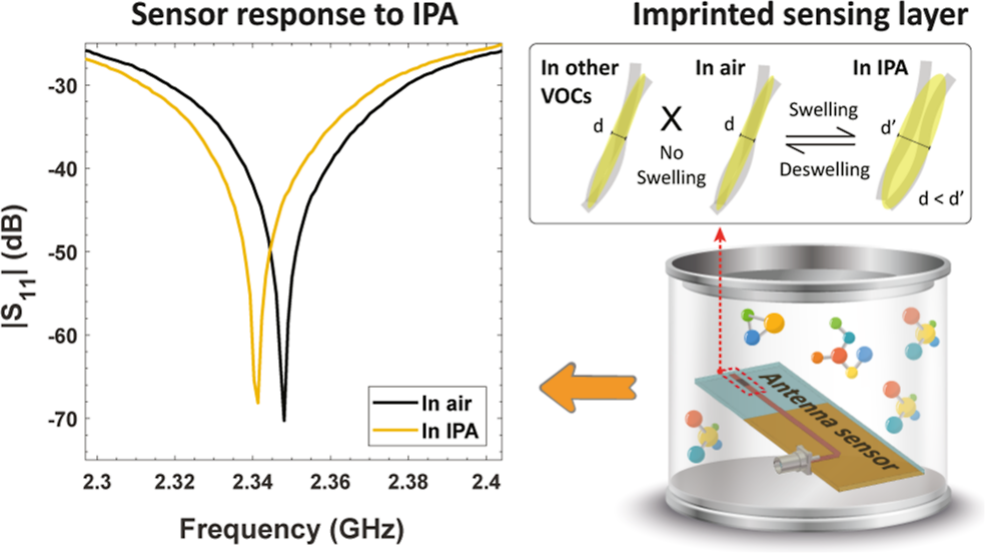

Accurate monitoring of isopropanol (IPA) levels is crucial for safety in industrial and laboratory settings, as high concentrations can lead to serious health issues. In this study, we present, for the first time, a dual-functional antenna sensor capable of high-performance IPA gas detection with concentration estimation and uninterrupted wireless communication, using optimized molecularly imprinted polymer (MIP)/multiwalled carbon nanotube (MWCNT)-based sensing materials. Comprehensive characterization of these materials confirms the successful formation and homogeneity of the composites. Furthermore, the electrical and gas-sensing properties of the sensing materials were evaluated using functionalized interdigitated electrode (IDE)-based sensing structures, optimized for high sensitivity, were functionalized to evaluate the electrical and gas-sensing properties of the materials. These IDE structures, which acted as impedance-varying components during operation, were coupled with a single-port monopole antenna to develop a highly sensitive and selective gas sensor while maintaining uninterrupted communication services. The results showed that the fabricated sensor platform exhibits strong selectivity, sensitivity, and stability for IPA detection at room temperature, effectively distinguishing it from other interference gases. In addition, using the same sensing material, we demonstrated that the antenna-based gas sensor exhibited higher sensitivity than the chemiresistive sensor, achieving a detection limit (18.8 ppm) below the safety thresholds for IPA. Moreover, the antenna’s radiation pattern and communication capabilities remained unaffected, ensuring uninterrupted functionality. Detailed optimization process and the sensing mechanism for a novel MIP-based selective antenna gas sensor, supported by both structural and electrical characterizations could serve as a milestone for future studies and the advancement of next-generation sensors.

The growing population and the integration of modern industry into daily life are making it increasingly difficult to maintain indoor air quality. Indoor environments, where people spend more than 85% of their time,^1^ can be up to ten times more polluted than outdoor air.^2^ Volatile organic compounds (VOCs) such as ethanol (EtOH), methanol (MeOH) and isopropanol (IPA), are significant indoor air pollutants.^3,4^ The Occupational Safety and Health Administration (OSHA) has established the permissible exposure limits (PEL) for these VOCs as 200 ppm for MeOH, 1000 ppm for EtOH, and 400 ppm for IPA. VOCs can be emitted from everyday sources such as chemicals, furniture, and even humans through exhaled breath, urine, and sweat.^5,6^ Due to their high vapor pressures, low molecular weights, and low boiling points, VOCs easily evaporate at room temperature (RT).^7^ The World Health Organization (WHO) has reported that exposure to VOCs poses significant risks to human health, including heart disease, stroke, diabetes, and lung cancer.^8^ Therefore, large-scale, accurate, sensitive, and selective detection of toxic VOCs at different locations is essential.

To date, a wide range of sensors have been developed for the sensitive and selective detection of VOCs. These systems rely on analytical reactions between the sensing material and the target gases, making the composition and quality of the sensing layers critical. Although various functional materials, including metal oxides, carbon-based materials, alloys, and organics, have been used as sensing layers, few exhibit sensitive, selective, and durable performance at RT. A promising technique involves using molecularly imprinted polymers (MIPs) as sensing layers.^9−11^

The selective detection capability of the MIP sensing layer for specific gases is achieved by a “lock and key mechanism,” where a template with the target analyte is used during synthesis.^12^ This process involves the formation of a complex of an imprint molecule and complementary polymerizable ligands.^13^ MIPs are synthesized by copolymerization of functional monomers and cross-linkers in the presence of templates.^14^ After removal of the template, cavities are created that match the size, shape, and functional groups of the templates, allowing MIPs to recognize template molecules.^15^ MIPs offer additional advantages, including ease of synthesis, low cost, and durability in harsh environments, making them highly suitable for practical applications.^16,17^ Moreover, these materials can be produced using environmentally friendly methods in line with green chemistry principles.^17^ These features make MIP-based materials valuable tools for the next-generation gas sensors.^18^

To enhance the electrical properties and surface-to-volume ratio of MIPs, they are often combined with conductive nanostructures, such as carbon nanotubes and metal nanostructures.^19^ For example, Jahangiri-Manesh et al. synthesized MIP/Au nanoparticle composites for VOC sensing.^6^ Cowen et al. developed MIP-multiwall carbon nanotube (MWCNT) composites as sensing layers for electrochemical gas sensing.^20^ Rong et al. created MIP-CNT composites for the selective detection of acetone gas.^21^ Zheng et al. synthesized SnO_2_-MIP composites for VOC detection. These studies confirm that MIP-based sensing layers have significant potential in gas sensing, although further research on material optimization and characterization is needed to advance their performance and applications. In these studies, sensing materials are typically analyzed for their electrical, electrochemical, and material properties to support traditional sensor development. However, antenna sensors operate using radio frequency (RF) signals—an area largely overlooked by traditional sensors. This research gap highlights the need for optimizing and characterizing MIPs to understand their sensing mechanisms and advance the development of antenna-based sensors.

In addition to the sensing layer, the choice of transducer is critical to gas sensor design.^22−24^ Commonly used transducers include optical, mass-sensitive, and chemiresistive types. However, these transducers often suffer from drawbacks such as high power consumption, and complex readout circuitry, which limit their mass deployment and integration with Wireless sensor networks (WSNs) and the Internet of Things (IoT). Microwave antenna-based transducers have emerged as promising candidates for next-generation sensor systems, effectively addressing these limitations while offering simplicity, low cost, low-powered and nonintrusive gas sensing at ambient conditions.^25−30^ When integrated with antennas, these sensors provide dual functionality—enabling both communication and sensing—making them ideal for WSNs.^25,29^ Hasan et al. developed a molybdenum disulfide-based microwave sensor for sensing applications, showing potential for methanol gas detection at RT.^31^ Wang et al. synthesized tin oxide/bionic carbon composites for microwave gas sensing devices, demonstrating sensitivity to ammonia at RT, unaffected by temperature and humidity variations.^26^ Ali et al. fabricated a microwave sensor device for gas sensing and reported sensitivity to acetone gas.^32^ Despite these advances, the lack of selectivity in microwave gas sensors limits their applications. Detailed optimization and material characterization, combined with appropriate antenna design, could lead to sensor platforms offering selective, sensitive, and durable VOC detection.

In this study, we developed a dual-functional antenna sensor for the first time, enabling simultaneous high-performance selective detection of IPA and communication. This was achieved through the synthesis of an MIP-based material, specifically designed to selectively detect IPA gas among VOCs, for which, to the best of our knowledge, no prior reports exist. MIP/MWCNTs heterostructures were synthesized and optimized using a simple green chemistry technique involving poly(vinyl alcohol), IPA, glutaraldehyde, and MWCNTs. Moreover, to design the antenna transducer element, the structural, electronic, and gas-sensing properties of the sensing layer must be examined in detail. The morphological, structural, and compositional properties of the MIP/MWCNTs composites were investigated using scanning electron microscopy (SEM), energy-dispersive spectroscopy (EDS), Fourier-transform infrared spectroscopy (FT-IR), and X-ray photoelectron spectroscopy (XPS). Interdigitated electrode (IDE) transducers were also employed to assess the electrical and gas-sensing properties of the MIP/MWCNTs. These IDE structures, which acted as impedance-varying components during operation, were coupled with a single-port monopole antenna to develop a highly sensitive and selective gas sensor while maintaining uninterrupted communication services. The results showed that the fabricated sensor platform exhibits strong selectivity, sensitivity, and stability for IPA detection at RT, effectively distinguishing it from other interference gases. Additionally, integrating the gas sensing structure with the monopole antenna preserved its radiation pattern and bandwidth, ensuring consistent wireless communication. These findings demonstrate that the developed device holds significant promise as a candidate for next-generation sensor systems. The step-by-step design and optimization procedure, along with the characteristics and sensing mechanism, offers a novel approach to developing application-specific MIP-based antenna sensors. Moreover, we provide a detailed explanation of the sensing mechanism for a MIP-based selective antenna gas sensor, supported by both structural and electrical characterizations. Consequently, these results could serve as a milestone for future studies and the advancement of next-generation sensors, applicable across a range of different applications.

## Methods

### Synthesis of MIP/MWCNTs Films

All chemicals used in this study, including poly(vinyl alcohol) (PVA), glutaraldehyde solution (50 wt % in water), and 2-propanol (≥99.5%), were obtained from Sigma-Aldrich (Merck). The MWCNTs were purchased from Nanocyl NC7000. First, 50 mg of PVA was dispersed in 3 mL of distilled water at 45 °C for 1 h using an ultrasonic bath. Then, three different volumes of IPA (2.5, 5, and 10 mL, referred to as MIP25, MIP50, and MIP100, respectively) were added to the prepared three solutions, which was stirred for 1 h at 45 °C. Next, 50 μL of glutaraldehyde, serving as a cross-linker, was added to the solutions and stirred for an additional 3 h at 85 °C. Simultaneously, a second solution was prepared by dispersing 8 mg of MWCNTs in 8 mL of IPA for each PVA solution in an ultrasonic bath at 60 °C for 2 h. Finally, these two solutions were combined and stirred at 45 °C overnight. The resulting materials were then coated onto IDE electrodes and antennas using simple drop-casting method. To optimize the sensing material, three sensors were synthesized for each of the three materials (MIP25, MIP50, and MIP100) with deposition volumes of 1.5, 3, and 6 μL, as shown in Table 1.

**Table 1:** Sample Codes and Synthesization Parameters

sample code	MWCNT (mg)	PVA (mg)	IPA (mL)	coating (μL)
MIP25_1	8	50	2.5	1.5
MIP25_2	8	50	2.5	3
MIP25_3	8	50	2.5	6
MIP50_1	8	50	5	1.5
MIP50_2	8	50	5	3
MIP50_3	8	50	5	6
MIP100_1	8	50	10	1.5
MIP100_2	8	50	10	3
MIP100_3	8	50	10	6

### Material Characterizations

The surface morphology and elemental composition of the synthesized materials were investigated using a Philips XL 30S SEM equipped with EDS. The functional groups of the synthesized materials were analyzed by FT-IR in the range of 500–4000 cm^–1^ (The PerkinElmer Spectrum 3). The chemical states and composition were examined by XPS using an Al Kα X-ray source (*h*ν = 1486.6 eV) and a hemispherical electron analyzer (Phoibos 150, SPECS GmbH). The basic pressure in the analysis chamber during the measurements was 3.7 × 10^–8^ Pa. The spectra were fitted using CasaXPS software with mixed Gaussian–Lorentzian peaks and subtraction of a Shirley-type background to extract the chemical bonding energies.

### Chemiresistive Sensors

**Figure 1:**
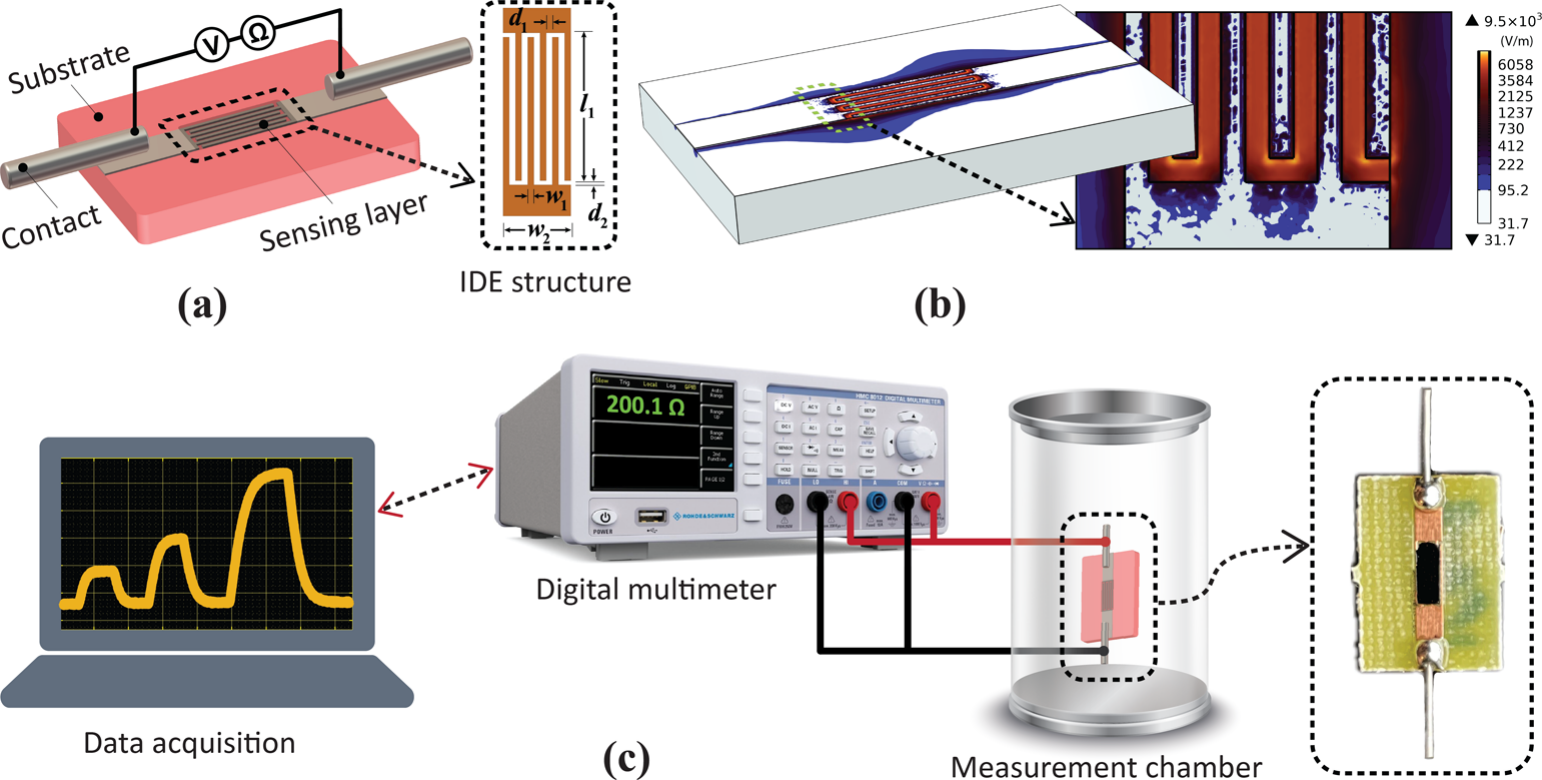
(a) CAD model illustrating the geometric parameters of the IDE-based chemiresistive sensor structure. (b) Electric field distribution within the optimized IDE structure, showing the deposited sensing material. (c) Experimental setup used to measure the chemiresistive gas sensor.

In this work, we used IDE-based transducers to optimize the sensitive material and perform its electrical characterization. The optimized sensing structure and material were later used in the development of the antenna sensor. The IDE structures were optimized for high-sensitivity detection by tuning the electrical coupling between the electrode fingers. This was achieved by adjusting design parameters such as the number of fingers, the width of the electrodes, and the gap between them. COMSOL Multiphysics was used to model, simulate and optimize the IDE structure, as shown in Figure 1a,b. To develop high-sensitivity chemiresistive transducers, both the sensing material and the IDE structure were optimized. In the simulation, a six-electrode copper structure (0.035 mm thick) was fabricated on an FR-4 PCB substrate, as shown in Figure 1a. Figure 1b illustrates the electric field distribution of the IDE structure with the sensing material as the material under test (MUT). Then, the designed IDEs were fabricated on copped coated side of FR4 substrates using a milling machine (LPKF ProtoMat S63).

Figure 1c illustrates the setup for chemiresistive gas sensing. Measurements were conducted using a digital multimeter (Rohde & Schwarz HMC8012) with a 4-wire resistance system. The sensor was placed in a custom-built 0.12-L gas chamber and connected to the multimeter to record a stable baseline resistance. Measurements were carried out under ambient conditions at RT (22 °C) with relative humidity between 63% and 66%. The sensors were exposed to VOCs at concentrations ranging from 1000 to 30,000 ppm by evaporating liquid VOCs into the chamber. Liquid VOCs were introduced into the chamber using a micropipette, with the amounts calculated based on the ideal gas law for methanol, ethanol, IPA, and acetone. Afterward, the chamber lid was closed. For recovery, the chamber lid was opened, and the system was allowed to sit until the sensor response returned to its initial baseline value. Dynamic changes in sensor resistance were monitored and recorded using LabVIEW software interfaced with the multimeter for data analysis. This change in resistance is detected by measuring the voltage across the IDE, allowing quantification of the gas concentration. This is represented by the absolute resistance values or the relative change in resistance, referred to as the sensor response (Δ*R*), which is calculated as follows
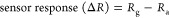
1where *R*_g_ is the resistance during gas exposure, and *R*_a_ is the initial resistance. The response time (τ_res_) and recovery time (τ_rec_) were determined as the time required for the sensor resistance to achieve 90% of its maximum variation during response and recovery, respectively.

### Antenna-Sensors

**Figure 2:**
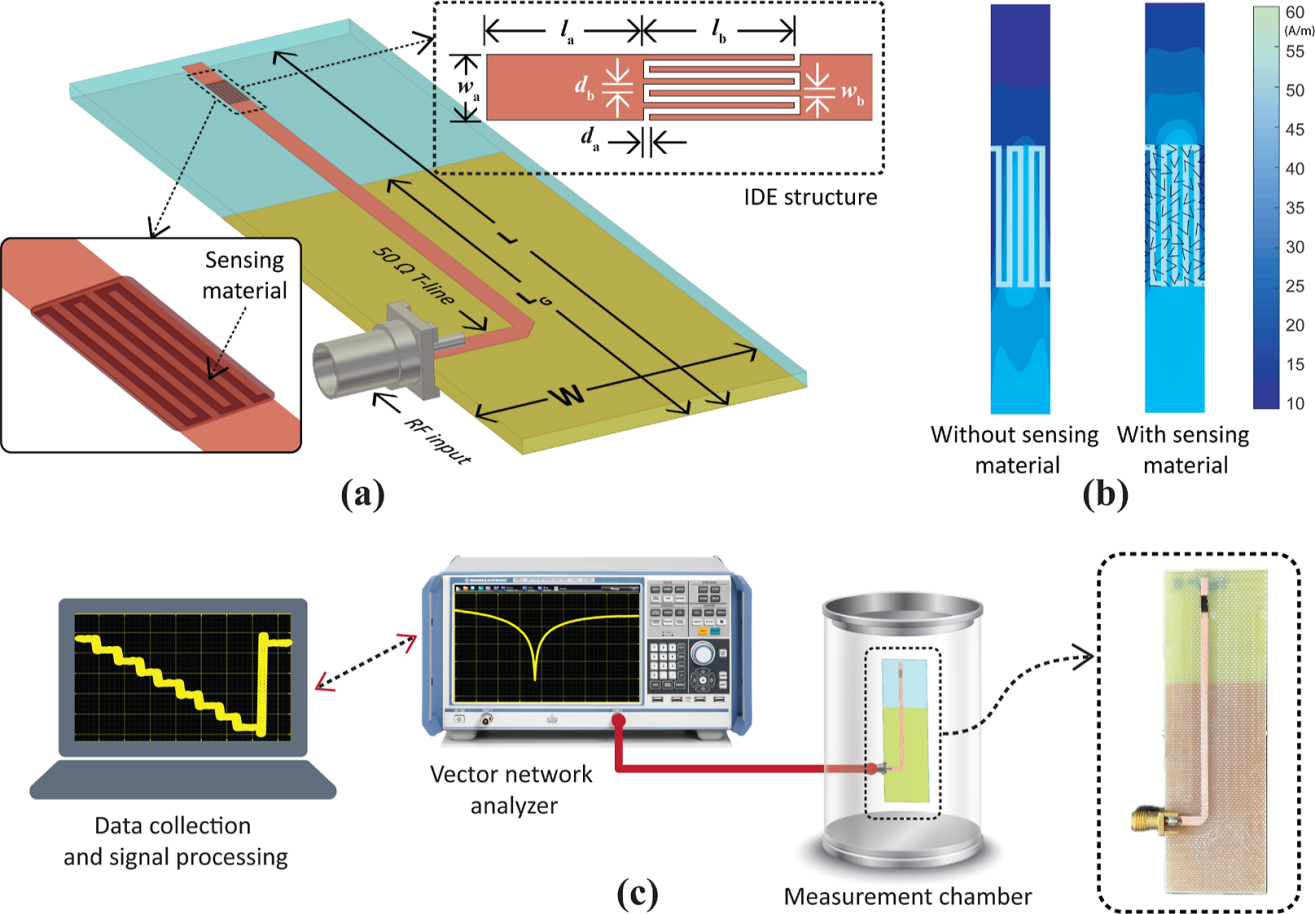
(a) 3D design and geometric parameters of the antenna sensor featuring the IDE structure. (b) Simulated surface current distribution of the optimized antenna’s sensing structure, shown with and without the sensing material. (c) Conceptual photograph of the experimental setup used to measure antenna-based gas sensors.

The primary objective of this research is to develop a dual-functional antenna sensor capable of both high-sensitivity, selective detection of IPA gas and continuous broadband wireless communication. To achieve this, a monopole antenna resonating at 2.45 GHz was first designed for wireless communication applications. The IDE structure was then incorporated into the antenna to introduce gas sensing capabilities, as illustrated in Figure 2a. The antenna was fabricated on a double-sided copper-coated FR4 substrate using an LPKF ProtoMat S63 milling machine, and connected with a 50 Ω SMA connector for signal excitation. It has dimensions of 89.5 × 30.57 mm, a relative permittivity of 4.7, a dielectric loss tangent of 0.02, and a thickness of 1.55 mm. The ground plane measures 61.63 mm in length, with a copper thickness of 0.035 mm.

The IDE structure was designed to maximize sensor response based on the properties of the sensing material. However, antenna sensors developed with this structure can exhibit significant frequency deviations during gas sensing, potentially detuning the antenna from its operating frequency and disrupting communication services. To address this issue, the IDE structures were reoptimized to control frequency shifts and ensure they remained within the desired operating bandwidth. Using the MUTs properties, the IDE structure was retuned using HFSS full-wave simulations to achieve the desired sensitivity. For example, an IDE sensor coated with MIP50_3 material showed an increase in dielectric constant from 103.5 to 110.2 when exposed to 1000 ppm IPA. Without further optimization, this change in dielectric constant could result in a resonance frequency shift of 3.5 MHz when integrated with an antenna sensor. However, by adjusting the geometric parameters of the sensing element, the sensor’s response can be adjusted to meet the requirements of specific applications. In our study, the optimized dimensions were *l*_a_ = 5.0 mm, *l*_b_ = 4.7 mm, *d*_a_ = 0.18 mm, *d*_b_ = 0.182 mm, *w*_a_ = 2.23 mm, and *w*_b_ = 0.22 mm.

The current distribution of the antenna with and without the sensing material is also illustrated in Figure 2b. The IDE structure, due to its interdigitated design, has inherent capacitive properties, resulting in minimal current flow through it and confining most of the current to the antenna’s main structure. However, when coated with the highly conductive sensing material, additional pathways for current flow are provided, allowing the current distribution to extend into the IDE structure. Therefore, the IDE structure carries more current, becoming part of the antenna’s sensing operation. This also increases the effective length of the antenna, causing shifts in the antenna’s resonance frequency. Although it is possible to further tune the antenna design parameters to retain the design frequency despite the deposition of the sensing material, this was not done in this study.

Antenna gas sensing was performed in a custom-built 5.2-L gas chamber as shown in Figure 2c. All *S*-parameter measurments, both before and after gas exposure, were made using a vector network analyzer (VNA, Rohde & Schwarz ZNB8). The VNA was configured to sweep from 1 to 4 GHz at 100 kHz intervals and the power level was set to −10 dBm. The antenna sensor was placed inside the gas chamber and connected to the VNA using an SMA cable. The baseline frequency of the antenna sensor was first recorded, followed by exposure to varying concentrations of VOCs ranging from 1000 to 8000 ppm. These concentrations were achieved by evaporating liquid VOCs inside the chamber, with the required quantities calculated using the ideal gas law. The liquid VOCs were introduced into the chamber using a micropipette, after which the chamber lid was sealed. For recovery, the lid was opened, and the system was left undisturbed until the sensor response returned to its baseline value. A stable resonance frequency was observed for each concentration before the sensor was exposed to the next concentration. Frequency shifts were measured relative to the baseline frequency, and sensor responses at all concentrations were recorded remotely using MATLAB. The sensor response for antenna sensors was calculated based on the frequency changes (Δ*f*). Similar to the chemiresistive sensors, the response time (τ_res_) and recovery time (τ_rec_) in the antenna sensor characterization were determined using the 90% change in the resonance frequency shift.

## Results

### Material Characterizations

**Figure 3:**
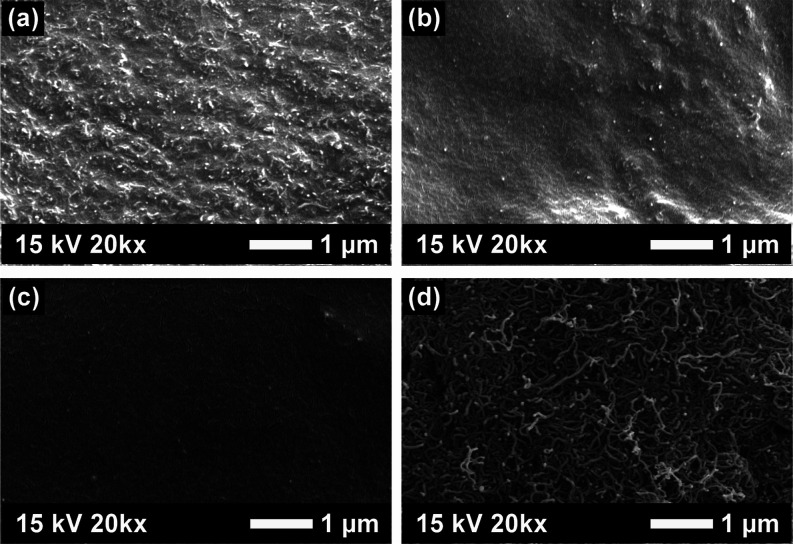
SEM images of the (a) MIP25, (b) MIP50, (c) MIP100, and (d) MWCNTs.

The chemical reactions that occur between the surface of a sensing layer and the target analyte make the study of surface morphology critical. The SEM images of the MIP/MWCNTs (a–c) and pristine MWCNTs (d) are presented in Figure 3.

It is observed that the MWCNTs are homogeneously distributed within the MIP and MIP/MWCNTs heterostructures are formed, as seen in Figure 3a–c. While MWCNTs can be clearly seen on the surface with the lowest amount of IPA (see Figure 3a), a homogeneous and smooth surface is obtained with increasing IPA during the synthesis, as seen in Figure 3c. In addition, the MIP25 sample, where MWCNTs are more visible, indicates that the MWCNTs are not well-incorporated into the MIP matrix, possibly due to the insufficient IPA amount.^33,34^

**Figure 4:**
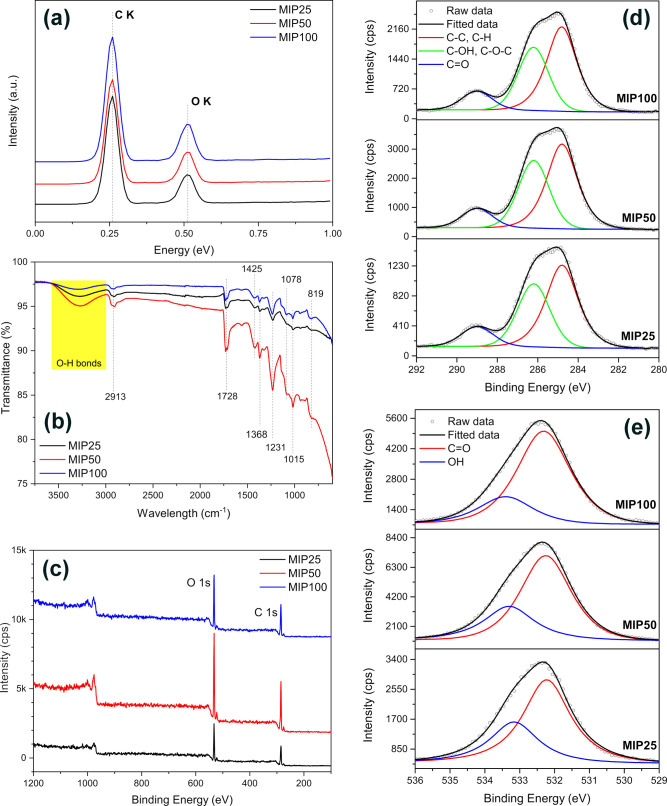
(a) EDS, (b) FT-IR, (c) XPS survey, (d) XPS C 1s and (e) XPS O 1s spectra of the MIP25, MIP50, and MIP100.

The EDS spectra of the synthesized materials are given in Figure 4a. The presence of C and O was observed in the spectra. An increase in the intensity of the O peak was also observed on the MIP/MWCNTs compared to the pristine MWCNTs. The C/O atomic ratios obtained by EDS analysis were 10.76, 2.2, and 2.2 for MIP25, MIP50, and MIP100, respectively. The higher C/O ratio in the MIP25 sample suggests a higher concentration of carbon (likely from MWCNTs) relative to oxygen (likely from the MIP). This may indicate a less efficient polymerization process, where more MWCNTs are exposed on the surface rather than being incorporated into the MIP structure.

The FT-IR spectra of the synthesized MIP-MWCNT films are presented in Figure 4b. The broad adsorption band observed at 3272 cm^–1^ corresponds to the hydroxyl groups –OH stretching vibration. The peak at 2913 cm^–1^ represents the asymmetric stretching vibration of the acrylic group (−CH_2_). A strong band at 1728 cm^–1^ indicates the characteristic C=O stretching in COOH groups. The adsorption peak at 1425 cm^–1^ corresponds to C–H bending, while the band at 1368 cm^–1^ is attributed to the O–H bending vibration in COOH groups. Additionally, peaks at 1231, 1078, 1015, and 819 cm^–1^ are attributed to C–C, C=O, C–O, and C–C–O stretching, respectively.^35−38^ The higher intensities of all peaks in MIP50, especially the –OH peak, may be related to the improved composite formation between PVA and MWCNTs.^39^

The chemical compositions and structure of MIP/MWCNTs heterostructures were investigated by XPS measurements. Figure 4c shows the XPS survey spectra of the synthesized materials. The two peaks can be associated to O 1s and C 1s and the atomic concentrations of carbon and oxygen were 69% and 31%, respectively, for all samples. The C 1s and O 1s core level spectra of the MIP/MWCNTs heterostructures are shown in Figure 4d,e. The C 1s core level spectra were deconvoluted into three major sources of carbon. The strongest peak at 284.8 eV is associated to hydrocarbon species (C–C/C–H). Another peak located at 286.2 eV is ascribed to C–O–C and C–OH bonds. The highest energy peak observed at 289 eV is associated to carboxylate carbon (O–C=O).^40−44^ The O 1s core level spectra were deconvoluted into two major sources of Oxygen, as seen in Figure 4e. The lowest energy at 532.2 eV peak related to the C–O/C=O bonds. The highest energy at 533.2 eV peak is ascribed to the hydroxyl groups (−OH).^45,46^ This result is in accordance with the FT-IR results.

### Chemiresistive Sensor Response

**Figure 5:**
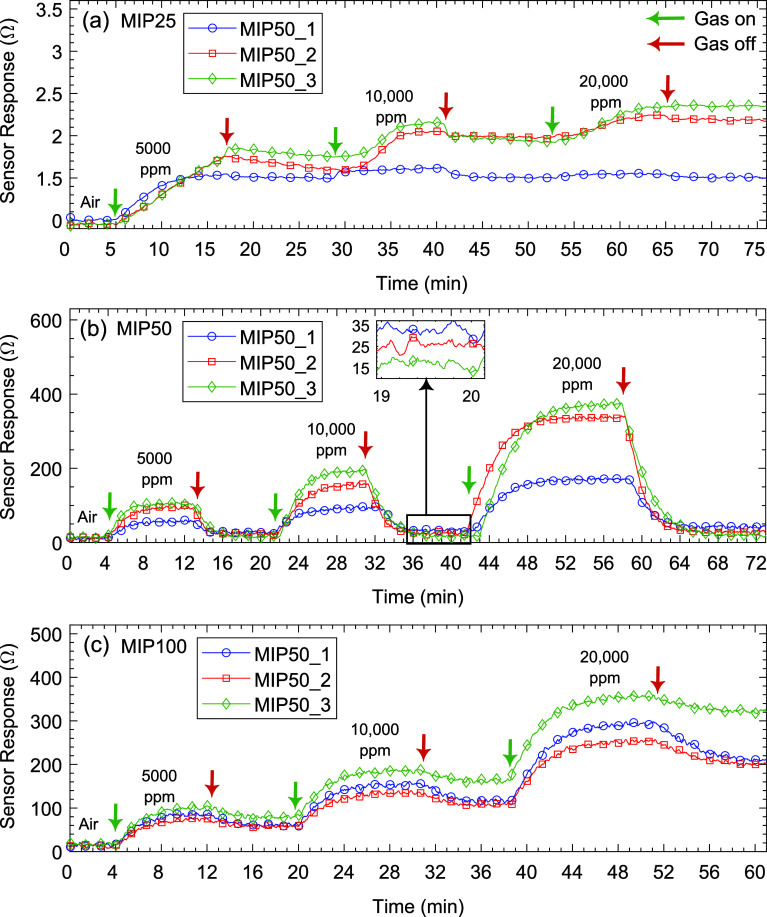
Sensor responses of (a) MIP25, (b) MIP50, and (c) MIP100 chemiresistive sensors to IPA.

To optimize the IDE structure, we first determined the properties of the MUT. The gaps between the electrodes were filled with the synthesized sensitive material and its electrical conductivity was measured as 0.43 × 10^–3^ S using a Rohde & Schwarz HM8118 LCR bridge meter. The impedance of the IDE was 2.3 kΩ – *j*128.5 Ω. The dielectric constants were measured using capacitance values before (*C*_0_) and after (*C*_d_) deposition of the sensing material. (*C*_0_) was 1.15 pF and *C*_d_ was 119.1 pF, giving a dielectric constant of 103.5. A DC voltage of 1.0 V was applied for the measurements. The IDE structure was then optimized to maximize the electric field strength, which was highest at the electrode edges and decreased toward the center of the gaps. The optimized parameters of the IDE structure are finger width *w*_1_ of 0.18 mm, distance between fingers *d*_1_ of 0.206 mm, finger length *l*_1_ of 4.8 mm, and IDE structure width *w*_2_ of 2.11 mm. Figure 5 presents the gas sensing performance of these nine sensors when exposed to IPA at concentrations of 5000, 10,000, and 20,000 ppm.

**Figure 6:**
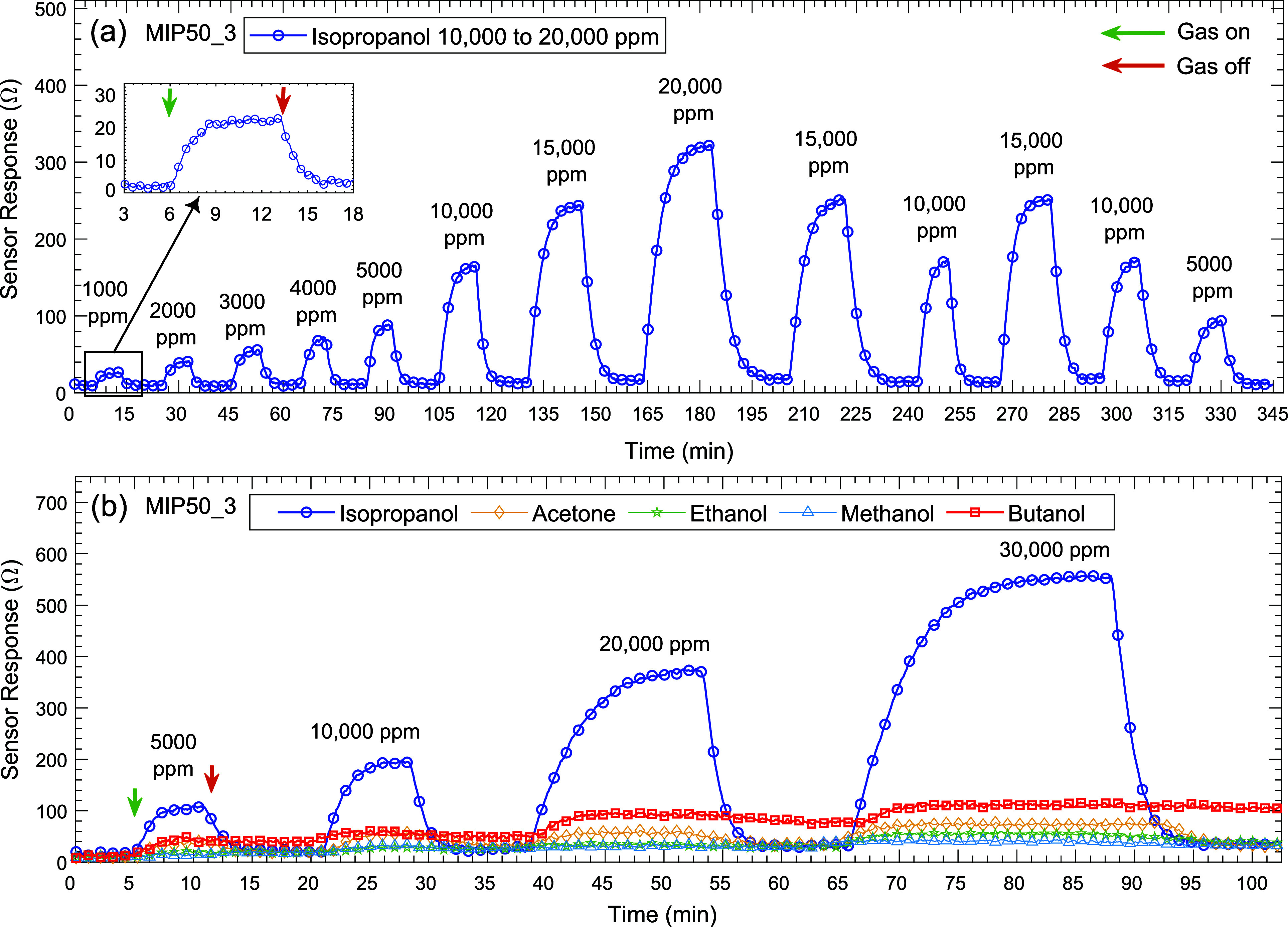
Dynamic sensor response of MIP50_3 sensor against (a) different concentrations of IPA, and (b) different concentrations of methanol, ethanol, IPA, butanol, and acetone.

Figure 5a shows that MIP_25_ sensors exhibited minimal resistance change up to 20,000 ppm IPA, with a response time of ∼6 to 8 min and no significant recovery. In Figure 5b, MIP50-based sensors demonstrated good sensing characteristics. All three variants of the MIP50-based sensors exhibited stable responses with a response time of ∼5.5 to 10.2 min and a recovery time of ∼3 to 6 min when exposed to IPA concentrations ranging from 5000 to 20,000 ppm. However, the MIP50_3-based sensor demonstrated better recovery compared to the other variants of this material. In Figure 5c, the MIP100_3-based sensors exhibited a response to IPA, but with longer response times of 8–12 min and partial recovery occurring within 6–10 min. Partial recovery can lead to inaccurate readings, data interpretation and potentially affect overall sensor performance. Chemiresistive sensor measurements reveal that the sample with a lower IPA concentration exhibits reduced sensitivity, weak recovery, and unclear sensor signals. Varying the IPA amount during synthesis influences the density and size of the imprinted cavities in the MIP.^47,48^ A higher IPA concentration promotes the formation of more imprinted cavities, enhancing the interaction strength between the cavities and IPA molecules, thereby improving sensor sensitivity and response. These findings are consistent with the FT-IR and EDS characterizations of the MIP-based sensing layers, which suggest that a lower solvent amount results in less incorporation of MWCNTs into the MIP matrix, likely due to insufficient IPA. This inadequate incorporation can disrupt the film structure and hinder the interaction between the sensor surface and the target analyte, leading to a weaker sensor signal. Therefore, the MIP50_3 sensor was selected for further experiments due to its superior performance. We then analyzed the performance of the MIP50_3-based sensor over a range of IPA concentrations, from low to high, and its selectivity against other interfering gases. In Figure 6a, the sensor demonstrated a response (Δ*R*) ranging from 21.5 to 362.5 Ω for isopropanol concentrations between 1000 and 20,000 ppm, respectively.

Recovery times ranged from 3.5 to 10.2 min, with negligible variations observed in repeatability tests over multiple measurements at the same concentrations. In Figure 6b, the MIP50_3 sensor demonstrated strong selectivity, sensitivity, and repeatability for IPA among other VOCs. This confirms that the synthesized MIP50_3 material is suitable for the development of a highly sensitive and selective IPA gas sensor, and an antenna sensor design was carried out based on this material.

### Antenna-Sensor Response

**Figure 7:**
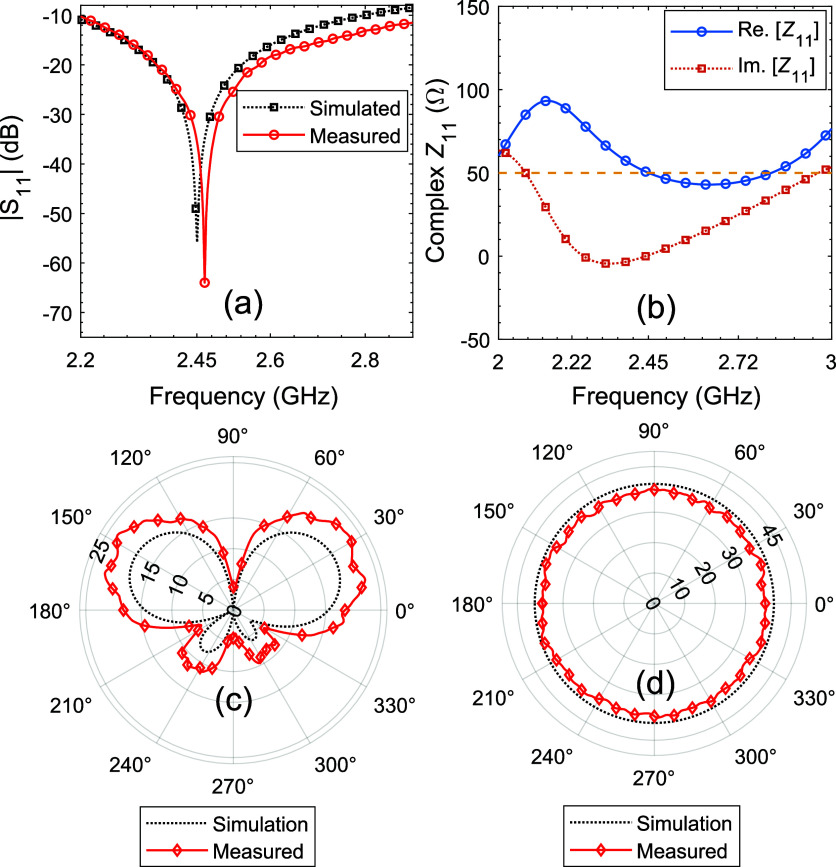
(a) Magnitude of *S*_11_ for the antenna sensor: simulated and measured before coating with sensing material. (b) Complex input impedance *Z*_11_ of the antenna. Simulated and measured radiation patterns without the sensing material in the (c) *E*(*yz*)-plane and (d) *H*(*xz*)-plane.

Prior to the gas sensing measurements, the design and parameters of the antenna platform were optimized. Figure 7a shows the simulated and measured *S*-parameters of the antenna, confirming a 10-dB bandwidth of 762 MHz (ranging from 2.209 to 2.971 GHz).

The difference between the measured (2.461 GHz) and simulated resonance frequency (2.45 GHz) is due to the fabrication tolerances of the milling machine (LPKF ProtoMat S63). Figure 7b shows the complex impedance (*Z*_11_), where the real part (resistance) represents the power radiation and the imaginary part (reactance) represents the stored energy. At 2.45 GHz the imaginary part of the impedance is zero (indicating resonance), and the real part is close to the characteristic impedance (*Z*_0_ = 50 Ω), ensuring maximum power transmission. This confirms that the antenna is perfectly matched at 2.45 GHz, efficiently radiating power with minimal reflection.^49−51^Figure 7c,d show simulated and measured radiation patterns of the antenna in the *E*(*yz*) and *H*(*xz*)-planes. The radiation pattern of the monopole antenna is omnidirectional, emitting an equal amount of power in all directions perpendicular to the antenna. This ensures uniform signal coverage, making it ideal for 360° communication in devices like mobile phones and Wi-Fi routers. However, due the finite size of the ground plane of the monopole antenna, the radiation pattern is tilted away from the horizontal plane. The monopole antenna achieves a peak gain of 3.92 dBi.

**Figure 8:**
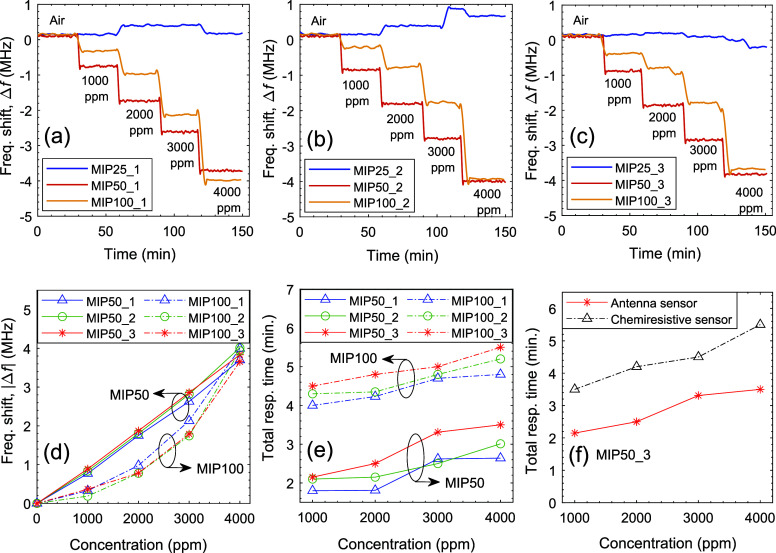
(a–c) Frequency shifts of MIP25, MIP50, and MIP100 coated antenna sensors upon exposure to different concentrations of IPA, (d) sensor response and (e) total response time of MIP50 and MIP100-based antenna sensors. (f) Comparison of response time between the MIP50_3-based antenna sensor and the MIP50_3-based chemiresistive sensor.

The gas sensing performance of MIP-coated antenna sensors was tested against IPA at concentrations ranging from 1000 to 4000 ppm. While the MIP25-coated sensors exhibited quite low and inconsistent frequency shifts, the MIP50- and MIP100-based antenna sensors demonstrated similar and reliable sensor responses in terms of frequency shifts (see Figure 8a–c). When the concentration-dependent frequency shifts are examined (see Figure 8d), MIP50 exhibits a more linear frequency shift with increasing concentration, particularly at lower concentrations. In contrast, MIP100 displays greater variation in frequency shift across the concentration range, which may suggest the presence of nonlinear interactions between the analyte and the MIP100 material. This observation implies that MIP50 achieves a more consistent interaction with the analyte over the tested concentration range. The improved linearity of MIP50 could be attributed to its structural properties, which may facilitate more uniform adsorption or binding of analyte molecules, resulting in a more predictable sensor response. Additionally, the MIP50-based antenna sensor achieved a stable response faster than the other materials, as shown in Figure 8. In Figure 8f, the total response time of the antenna-based sensor is compared with that of a chemiresistive sensor, with the antenna sensor showing a significantly faster response, approximately 2 min faster.

**Figure 9:**
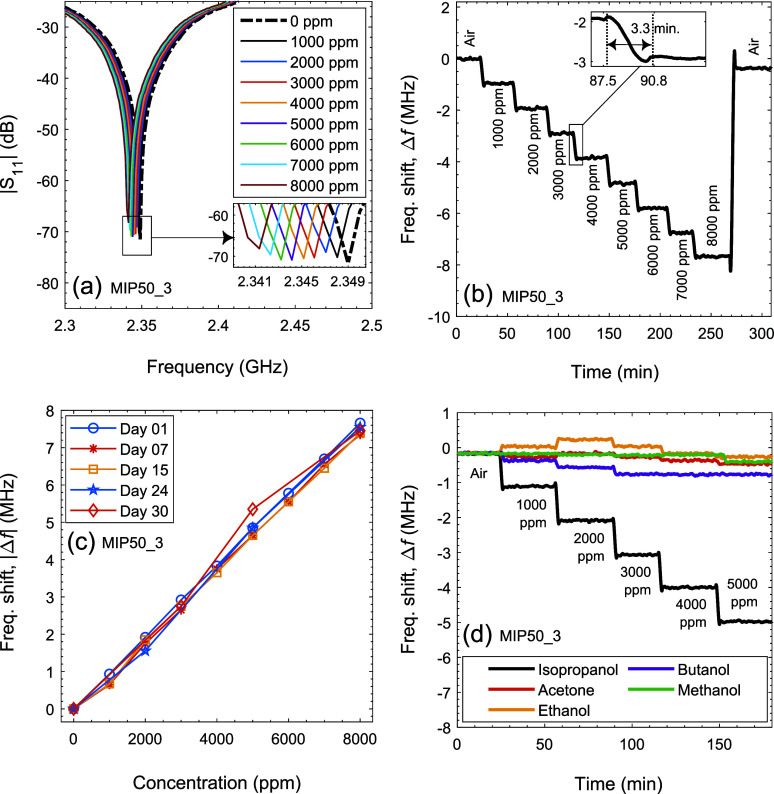
(a) *S*_11_ values as a function of IPA concentration, (b) time-dependent frequency shifts during gas exposure, with an inset showing response time, (c) sensor’s reliability test over 30 days, and (d) selectivity test of the MIP50_3-coated antenna sensor.

It should be noted that coating the IDE fingers with the sensing layer creates an interconnecting path between the electrodes, which increases the effective length of the antenna and shifts its resonant frequency to 2.349 GHz, as shown in Figure 9a. This becomes the baseline frequency of this antenna sensor and all frequency shifts have been calculated in relation to this value.

The resonance frequency of the MIP50_3-based antenna sensor shifted from 2.3490 to 2.3481 GHz at 1000 ppm IPA exposure, indicating a clear sensor response of ∼1 MHz (see Figure 9a). Higher concentrations resulted in similar stable frequency shifts of around 1.0 MHz per 1000 ppm. The dynamic, time-dependent frequency signal of the antenna sensor is presented in Figure 9b. The sensor exhibited a clear and proportional response across a concentration range from 1000 to 8000 ppm, as evidenced by distinct frequency shifts. During the recovery phase, the sensor returned to its baseline frequency, demonstrating excellent reversibility and reusability. While similar performance has been reported in other antenna-based gas sensor studies,^52−55^ the selective, sensitive, and stable frequency shift observed in this study represents a significant advancement in the field. Furthermore, the antenna sensor exhibited a rapid response time, with stabilization typically achieved within 3–3.5 min, as highlighted in the inset of Figure 9b. A distinct linear relationship was observed in Figure 9c. To ensure the reliability of the sensor, multiple experiments were conducted over 15 days to account for environmental variations and potential aging of the sensing material. These tests consistently produced comparable results under the same conditions, as shown in Figure 9c. During the selectivity tests for the MIP50_3-based antenna sensor, it was exposed to acetone, ethanol, methanol, and butanol at concentrations ranging from 1000 to 5000 ppm (see Figure 9d). The sensor showed negligible to no response to these other gases, whereas it continued to detect IPA concentrations linearly.

**Figure 10:**
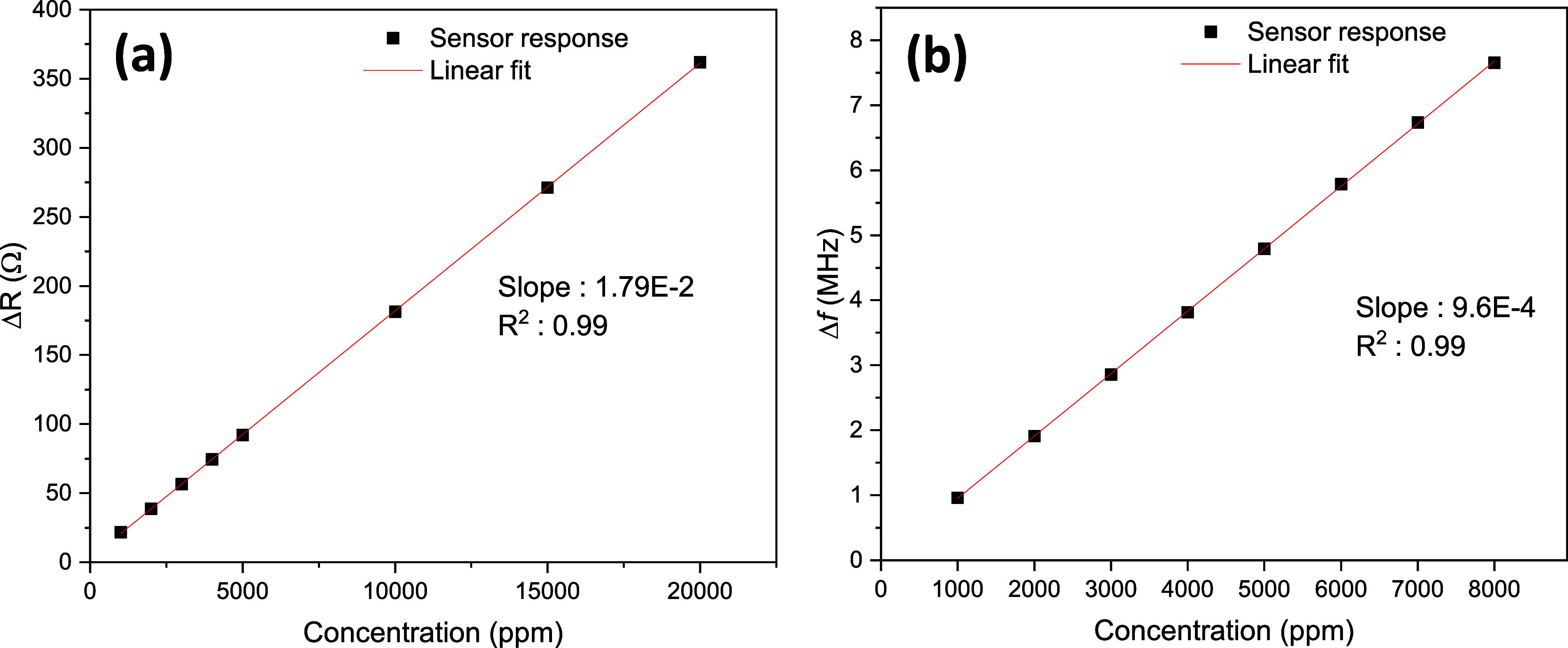
Calibration curves of MIP50_3-based (a) chemiresistive and (b) antenna sensor against IPA gas.

To better understand sensor performance, the minimum detectable concentration can be determined as a theoretical estimate derived under controlled experimental conditions. The semiempirical limit of detection (DL) was calculated for the MIP50_3-coated antenna and chemiresistive sensors to determine the lowest gas concentration they can reliably detect using signal processing techniques^56−58^
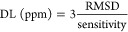
2where RMSD is the root-mean-square deviation (sensor noise) based on the curve-fitting equation. Sensitivity values were calculated using the linear fitting of sensor responses: Δ*R* for the chemiresistive sensor and Δ*f* for the antenna sensor (see Figure 10). It is clearly evident that both sensors exhibited linear responses to IPA gas. The detection limit of the antenna sensor is 18.8 ppm, while that of the chemiresistive sensor is 297 ppm, indicating the higher sensitivity of the antenna sensor.

Table 2 compares the sensing performance of the MIP-based antenna gas sensor with recently reported IPA sensors. It is evident that the MIP/MWCNT-coated antenna sensor has relatively high sensing performance for IPA gas, especially in terms of power consumption and selectivity. While existing IPA sensors often require high operating temperatures, the proposed sensor platform operates at RT, resulting in low power consumption. Furthermore, unlike most sensors that struggle to distinguish IPA from similar molecules (e.g., EtOH, MeOH), the MIP-based antenna sensor demonstrates selective behavior toward IPA.

**Table 2:** Comparison of IPA Gas Sensors

material	transducer	sensitive to	temp. (°C)	conc. (ppm)	ref
Ce–In_2_ O_3_	chemiresistive	IPA, ACE, MeOH, EtOH	220	100	(59)
Bi_2_ MoO_6_	chemiresistive	IPA, *n*-BuOH, EtOH	270	5	(60)
NiO	chemiresistive	IPA, EtOH, Toluol	100	60	(61)
Ho-ZnO	chemiresistive	IPA, *n*-PrOH, ACE	140	100	(62)
CuO/SnO_2_@Ag	chemiresistive	IPA, EtOH, ACE	200	100	(63)
MoO_3_	chemiresistive	IPA, EtOH	200	500	(64)
ZnFe_2_ O_4_	chemiresistive	IPA, EtOH	240	100	(65)
TiO_2_	chemiresistive	IPA, MeOH	50 + UV	50	(66)
ZIF-8/GO	fiberoptic	IPA, MeOH, EtOH	RT	1500	(67)
MIP/MWCNTs	microwave	IPA	RT	1000	this work

**Figure 11:**
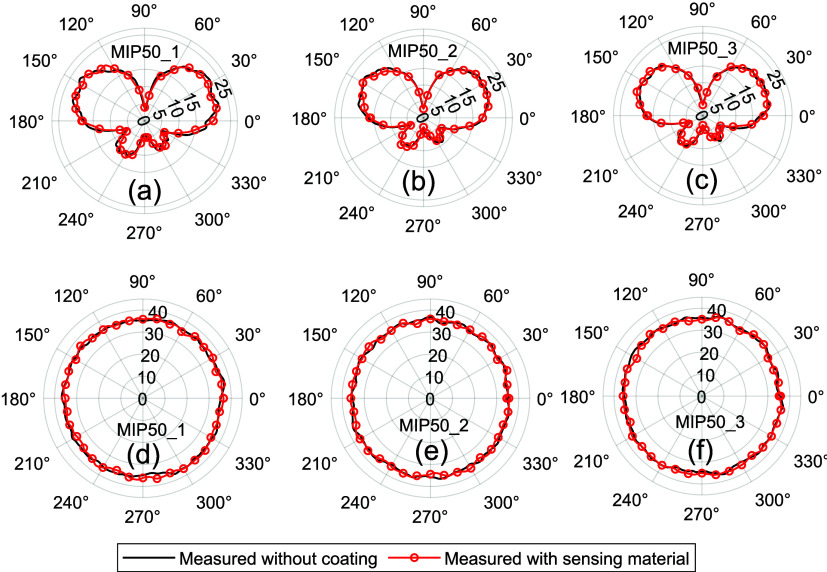
(a) Measured radiation patterns with and without the sensing material in the (a–c) *E*(*yz*)-plane and the (d–f) *H*(*xz*)-plane.

In addition to selective isopropanol detection, reliable wireless communication is critical to the dual functionality of the antenna sensor. The optimized sensor design and controlled frequency shifts provide wideband operability (762 MHz bandwidth), enabling flexible gas sensing (see Figure 7a). Maintaining the antenna radiation pattern is another key element for consistent signal coverage and performance. As shown in Figure 11a–f, coating the monopole antenna with the sensing layer does not affect its radiation pattern, ensuring uninterrupted communication. Therefore, a dual-functional antenna sensor for both high-performance gas sensing and reliable communication has been successfully developed.

### Sensing Mechanism

**Figure 12:**
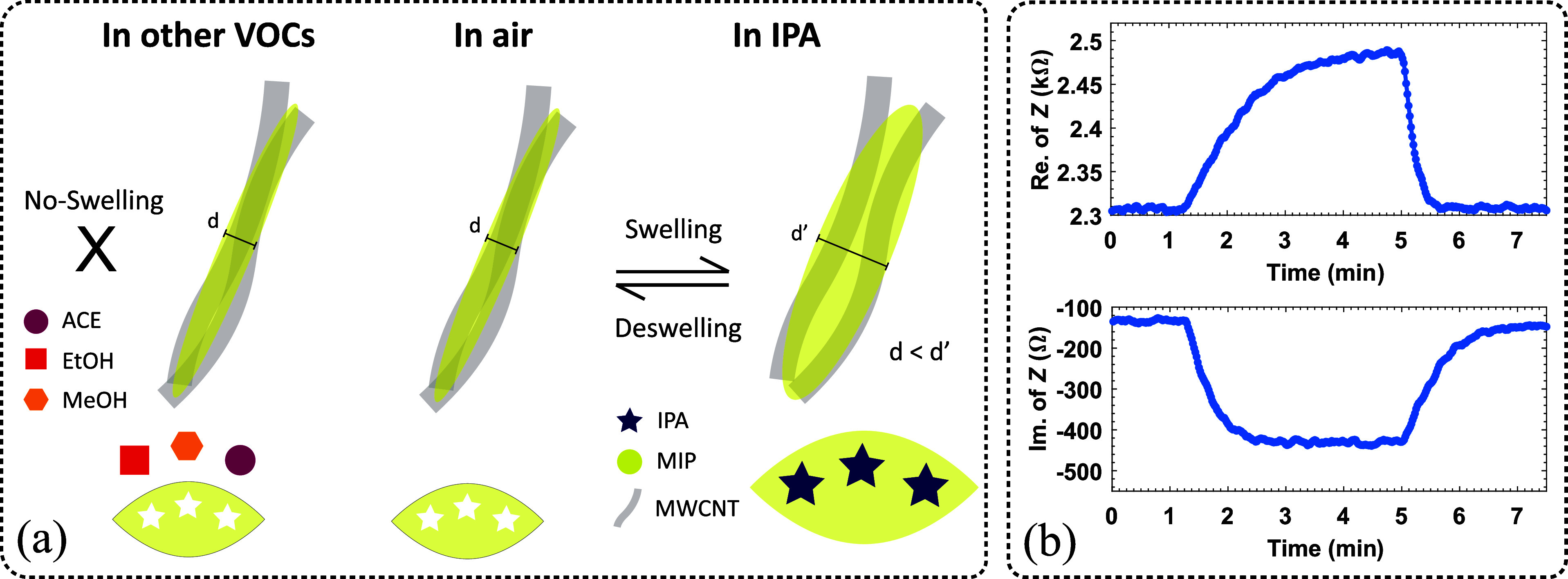
(a) Schematic illustration of the sensing mechanism for MIP/MWCNTs gas sensor. (b) Impedance response of the IDE structure to 10,000 ppm IPA, showing changes in the real and imaginary parts of the impedance.

The sensing mechanism of MIP/MWCNT sensors is likely to involve the swelling of the polymer matrix, as illustrated in Figure 12.^68−70^ This is a thermodynamic phenomenon where the polymer swells leading to volume expansion due to VOC adsorption.^71,72^ The adsorption primarily occurs between the VOCs and the MIP through hydrogen bonds (OH groups), and the swelling of PVA is proportional to the diffusion of VOCs.^71,73^ When VOCs are adsorbed by the PVA/MWCNT composite, the swelling of PVA increases the volume of the polymer, which in turn increases the distances between the randomly oriented nanotubes, which act as conductive pathways in the material.^69,74^ As a result, the contact resistance of the MWCNT network in the composite increases.^68^ The increase in resistance is proportional to the concentration of IPA gas, and after the recovery process, the resistance returns to the baseline value, as seen in Figure 5a. This confirms that the swelling mechanism is effective. The selective behavior toward IPA among the VOCs can be attributed to the molecular recognition properties of MIP. During synthesis, cavities are formed in the PVA matrix due to the removal of the template molecule. These imprints in the polymer network enable the selective rebinding of molecules with similar size, shape, and functional groups to the template molecule.^75,76^

The integration of the IDE design into the antenna platform allows changes in the electronic properties of the sensing layer to be detected by changes in the resonant frequency. When antennas were coupled to MIP/MWCNT-coated IDE structures, the impedance of the sensing element was also coupled to the load impedance of the antenna (*Z*_L_), causing a change in the reflection coefficient (*S*_11_) and a shift in the resonance frequency, as shown in Figure 9a. This can be explained by the mathematical expressions as^77,78^
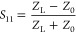
3

And
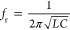
4where *L* and *C* represent inductance and capacitance of the antenna. During gas exposure, the effective permittivity of the dielectric medium (MIP/MWCNT) between the IDE fingers changes, resulting in changes in capacitance. As shown in Figure 12b, exposure to IPA changes both the real (resistive) and imaginary (reactive) components of the impedance of the MIP/MWCNTs (MIP5_3)-coated sensor, thereby shifting the resonance frequency of the antenna as described by eqs 3 and 4. Therefore, the MIP/MWCNT-coated IDE-based sensing structure functions as a variable impedance component that selectively responds only to IPA gas. This frequency shift can be used to accurately and selectively indicate the presence and concentration of the gas and is the core principle of the antenna-based gas sensor. Integrating this passive component into the antenna system creates an antenna-sensor with a resonance frequency that shifts with the IPA gas concentration. However, the amount of frequency shift with gas exposure can be controlled for specific wireless applications by optimizing the IDE structure, enabling selective continuous gas sensing and uninterrupted communication services simultaneously.

## Conclusions

In this study, we demonstrated, for the first time, the fabrication and application of a MIP/MWCNT-based dual-functional antenna sensor capable of simultaneous communication, and highly selective and sensitive detection, along with concentration estimation of IPA gas. The MIP matrix was designed and optimized to selectively bind IPA molecules. To enhance the electrical and sensing performance, a MIP/MWCNTs heterostructure was developed. These materials were characterized by various techniques including SEM, EDS, FT-IR, and XPS. SEM images confirmed the homogeneous distribution of MWCNTs within the MIP, while EDS analysis revealed the presence of C and O elements, with the C/O ratio decreasing as the amount of IPA increased. FT-IR spectra indicated successful composite formation between MIP and MWCNTs, and XPS revealed two major oxygen sources on the surface, namely C–O/C=O and –OH bonds. The MIP-based materials were applied in varying amounts to IDE-based chemiresistive transducers using the drop-casting method. Optimization of the IDE structures improved the electric field strength and enhanced the sensing performance. Chemiresistive measurements showed that MIP optimization by adjusting the amount of template molecule resulted in strong selectivity, sensitivity, and durability in the detection of IPA among other VOCs at RT. Optimizing the IDE structures enabled integration with a monopole antenna to control frequency shifts and maintain resonance within the operational bandwidth. Gas sensing measurements with the antenna sensor showed a linear response with high sensitivity and strong selectivity for IPA over other gases such as methanol, ethanol, butanol, and acetone. FT-IR and EDS analyses further support these findings, highlighting the significant role of solvent amount in determining the structural and functional properties of the sensing layer. The observed selectivity is attributed to the optimization of the imprinted cavities within the MIP layer, achieved by precisely controlling the IPA concentration during synthesis. A higher IPA concentration facilitated the formation of denser and more well-defined imprinted cavities, enhancing the interaction strength between the cavities and IPA molecules. In contrast, a lower IPA concentration resulted in fewer imprinted cavities, a disrupted film structure, and reduced incorporation of MWCNTs into the MIP matrix, ultimately leading to weaker sensor signals. The antenna sensor has a detection limit of 18.8 ppm and a response time of ∼2 min, outperforming the chemiresistive sensor’s ∼297 ppm detection limit and 3.5 min response time. The 30-day aging test confirmed the stability of the sensors. Moreover, coating the monopole antenna with the sensing layer did not affect its radiation pattern, ensuring uninterrupted communication. Therefore, MIP/antenna heterostructures represent a powerful sensor platform for high-performance gas detection and reliable communication. We believe that this novel approach, supported by detailed design techniques, material characterization, and sensing mechanisms, represents a significant advance in sensor technology.
